# Surface characterization of an ultra-soft contact lens material using an atomic force microscopy nanoindentation method

**DOI:** 10.1038/s41598-022-24701-9

**Published:** 2022-11-21

**Authors:** Vinay Sharma, Xinfeng Shi, George Yao, George M. Pharr, James Yuliang Wu

**Affiliations:** 1 Alcon Research, LLC, Fort Worth, TX 76134 USA; 2 Alcon Research, LLC, Duluth, GA 30097 USA; 3 Department of Materials Science and Engineering, Texas A&M University, College Station, TX 77843 USA

**Keywords:** Analytical chemistry, Materials chemistry, Polymer chemistry, Surface chemistry, Biomaterials, Soft materials

## Abstract

As new ultra-soft materials are being developed for medical devices and biomedical applications, the comprehensive characterization of their physical and mechanical properties is both critical and challenging. To characterize the very low surface modulus of the novel biomimetic lehfilcon A silicone hydrogel contact lens coated with a layer of a branched polymer brush structure, an improved atomic force microscopy (AFM) nanoindentation method has been applied. This technique allows for precise contact-point determination without the effects of viscous squeeze-out upon approaching the branched polymer. Additionally, it allows individual brush elements to be mechanically characterized in the absence of poroelastic effects. This was accomplished by selecting an AFM probe with a design (tip size, geometry, and spring constant) that was especially suited to measuring the properties of soft materials and biological samples. The enhanced sensitivity and accuracy of this method allows for the precise measurement of the very soft lehfilcon A material, which has an extremely low elastic modulus in the surface region (as low as 2 kPa) and extremely high elasticity (nearly 100%) in an aqueous environment. The surface-characterization results not only reveal the ultra-soft nature of the lehfilcon A lens surface but also demonstrate that the elastic modulus exhibits a 30 kPa/200 nm gradient with depth due to the disparity between the modulus of the branched polymer brushes and the SiHy substrate. This surface-characterization methodology may be applied to other ultra-soft materials and medical devices.

## Introduction

The mechanical properties of materials developed to be used in direct contact with living tissues are often dictated by the surrounding biological environment. An ideal overlap of these materials properties helps to achieve the desired clinical performance of the material without prompting undesirable cellular responses^1–3^. For bulk, homogenous materials, characterization of mechanical properties is relatively straightforward due to the availability of standard testing techniques and methods such as microindentation^4–6^. However, for ultra-soft materials such as gels, hydrogels, biopolymers, living cells, etc., these testing methods are often not applicable because of measurement-resolution limitations as well as the heterogenous nature of some of the materials^7^. Over the years, traditional indentation methods have been modified and customized in order to characterize a wide range of soft materials, but many still suffer from severe drawbacks that limit their use^8–13^. This lack of specialized testing methods that can accurately and reliably characterize the mechanical properties of ultra-soft materials and surface layers significantly limits their potential use in a wide variety of applications.

In our previous work, we presented the lehfilcon A contact lens (CL)—a soft, heterogeneous material that derives all of its ultra-soft surface properties from the underlying biomimetic design, inspired by the ocular corneal surface^14^. This biomaterial was engineered by grafting a branched, crosslinked poly (2-methacryloyloxyethyl phosphorylcholine (MPC)) (PMPC) polymeric layer to a medical-device-grade silicone hydrogel (SiHy) base substrate^15^. This grafting process produces a layer comprising a very soft and highly elastic, branched polymer-brush structure at the surface. Our earlier work established that the biomimetic structure of the lehfilcon A CL provides exceptional surface properties, such as enhanced wettability and antifouling behavior, increased lubricity, and reduced cell and bacterial adhesion^15,16^. Moreover, the use and engineering of this biomimetic material also suggest further extensions applicable to other biomedical devices. Therefore, it is critical to characterize the surface properties of this ultra-soft material and understand its mechanical interaction with the eye, in order to develop a comprehensive knowledge base to support future developments and applications. The majority of the commercially available SiHy contact lenses are composed of a homogenous mixture of hydrophilic and hydrophobic polymers that form a uniform material structure^17^. Several studies have been conducted to examine their mechanical properties using conventional compression, tensile and microindentation testing methods^18–21^. However, the novel biomimetic design of the lehfilcon A CL makes it an unique heterogenous material, in which the mechanical properties of the branched, surface polymer-brush structure are significantly different from those of the underlying SiHy base substrate. As a result, it is very challenging to accurately quantify these properties using traditional indentation techniques and methods. One promising technique makes use of nanoindentation testing methods implemented in an atomic force microscope (AFM)—a technique that has been used to determine the mechanical properties of soft viscoelastic materials such as biological cells and tissues, as well as soft polymers^22–30^. In AFM nanoindentation, the fundamentals of nanoindentation testing are combined with recent advances in AFM technology to provide both higher measurement sensitivity and the capability to test a wide range of inherently ultra-soft materials^31–36^. In addition, the technique offers other important advantages through the use of multiple indenter-probe geometries and the ability to test in different liquid environments.

AFM nanoindentation can be broadly divided into three main components: (1) hardware (sensors, detectors, probe etc.); (2) measurement parameters (e.g., force, displacement, speed, ramp size, etc.); and (3) data processing (baseline correction, contact point estimation, data fitting, modelling etc.). One outstanding issue with the technique is that some studies in the literature that use AFM nanoindentation report very dissimilar quantitative results for the same sample/cell/material type^37–41^. For example, Lekka et al. studied and compared the effects of AFM probe geometries on the measured Young’s moduli of mechanically homogenous hydrogels and heterogenous cell samples. They reported that the modulus values were highly dependent on the choice of cantilevers and the tip shape, with pyramidal probes giving the highest values and spherical probes the lowest^42^. Similarly, Selhuber-Unkel et al. have shown how the indenter speed, indenter size and thickness of polyacrylamide (PAAm) samples can all influence Young’s moduli measured using AFM nanoindentation^43^. An additional complication is the unavailability of standard testing materials with very low elastic modulus and complimentary testing techniques. This makes it very challenging to obtain accurate results with confidence. Nevertheless, the method is extremely useful in conducting relative measurements and comparative assessments between similar sample types, for example, using AFM nanoindentation to discriminate between normal and cancerous cells^44,45^.

When testing a soft material with AFM nanoindentation, a general rule of thumb is to use a probe with a low spring constant (*k*) that closely matches the modulus of the sample and a hemispherical/rounded tip so that at the first contact with the soft sample, the probe does not pierce through the sample surface. It is also important that the probe generates a deflection signal that is high enough to be recorded by the laser detector system^24,34,46,47^. In the case of ultra-soft heterogenous cells, tissues, and gels, an additional challenge is overcoming the adhesive forces between the probe and the sample surface in order to assure reproducibility and reliability of the measurements^48–50^. Until very recently, most AFM nanoindentation work that focused on studying the mechanical behavior of biological cells, tissues, gels, hydrogels and biomolecules involved the use of relatively large spherical probes commonly called as colloidal probes (CP)^26,43,47,51–55^. The radius of these probes can vary from 1 to 50 µm, and they are usually made up of borosilicate glass, polymethyl methacrylate (PMMA), polystyrene (PS), silicon dioxide (SiO_2)_ and diamond-like-carbon (DLC). Even though CP-AFM nanoindentation is often the preferred choice for the characterization of soft samples, it has its own challenges and limitations. The use of a large, micrometer-sized spherical tip increases the overall tip-sample contact area and causes significant reduction in the spatial resolution. For a soft, heterogenous sample, where the mechanical properties of the local features can be remarkably different from the average over a wider area, CP indentation can lead to obscuring of any heterogeneity in the properties on a local scale^52^. Colloidal probes are usually fabricated by attaching micrometer-size colloidal spheres to tipless cantilevers with epoxy-based adhesives. The fabrication process itself presents many challenges and can cause inconsistencies during calibration of the probes. In addition, the size and the mass of the colloidal particle directly impact the primary calibration parameters of the cantilever such as the resonant frequency, spring constant and the deflection sensitivity^56–58^. Therefore, the usual methods such as thermal-tune calibration used for conventional AFM probes may not provide accurate calibration for CP, and additional methods may be required to perform these corrections^57,59–61^. Typical CP indentation experiments use large cantilever deflections to examine the properties of soft samples, and this produces another challenge for calibrating the non-linear behavior of the cantilever at relatively large deflections^62–64^. Current colloidal-probe indentation methods often consider the geometry of the cantilever for probe calibration but neglect the effects of the colloidal particle, producing additional uncertainties in the accuracy of this method^38,61^. Similarly, calculations of the elastic modulus by the fitting of contact models are directly dependent on the geometry of the indenting probe, and a mismatch between the tip and a sample surface feature may lead to innaccuracies^27,65–68^. Some recent work by Spencer et al. underscores the factors to be considered when using CP-AFM nanoindentation methods to characterize soft polymer brushes. They reported that rate-dependent, viscous-fluid confinement within polymer brushes can lead to increased indenter loads and therefore give rise to apparent rate-dependent property measurements^30,69–71^.

In this study, we characterized the surface modulus of an ultra-soft, highly elastic material, lehfilcon A CL, using an improved AFM nanoindentation method. Considering the properties and the novel structure of this material, it was clear that the sensitivity range of traditional indentation methods would be inadequate to characterize the modulus of such an extremely soft material, and it would therefore be necessary to use an AFM nanoindentation technique with higher sensitivity and lower noise levels. After reviewing the inadequacies and problems of existing colloidal-probe AFM nanoindentation methods, we show why we selected a specially designed smaller AFM probe that addresses the issues of sensitivity, background noise, precise contact-point determination, rate-dependent fluid confinement and accurate quantitative modulus measurements for soft heterogenous materials. In addition, our ability to measure the shape and size of the indenting tip accurately allowed us to use a cone-sphere fitting model that enables the determination of elastic modulus without the necessity of estimating the tip-material contact area. Two implied assumptions that were made to conduct quantitative assessments in this work are fully elastic nature of the material and the modulus being independent of the indentation depth. Using this approach, we first tested an ultra-soft standard sample of known modulus to quantitatively evaluate the method, and then used the method to characterize the surface of two different contact-lens materials. This AFM nanoindentation surface-characterization methodology with enhanced sensitivity is expected to be applicable for a wide range of biomimetic heterogenous, ultra-soft materials that are of potential use in medical-device and biomedical applications.

## Materials and methods

### Materials

A lehfilcon A contact lens (Alcon, Fort Worth, Texas, USA) and its silicone hydrogel base substrate were selected for the nanoindentation experiments. A custom-designed lens holder was used for the experiments. To mount the lens for testing, it was carefully positioned on a dome-shaped holder, making sure that no air bubbles were entrapped, and then clamped in place by the edges. An opening in the top fixture of the lens holder provided access to the lens optical center for nanoindentation experiments, while at the same time holding the fluid in place. This maintained the lens in a fully hydrated condition. 500 µl of the contact lens packaging solution was used as the testing fluid. To validate the quantitative results, a commercially available, non-activated polyacrylamide (PAAm) hydrogel composed of polyacrylamide-co-methylene-bis-acrylamide (Petrisoft 100 mm dish, Matrigen, Irvine, California, USA) with a known elastic modulus of 1 kPa was tested. Testing on the Petrisoft hydrogel was conducted using 4–5 drops (~ 125 µl) of phosphate-buffered saline (PBS from Corning Life Sciences, Tewksbury, Massachusetts, USA) and 1 drop of OPTI-FREE Puremoist contact-lens solution (Alcon, Fort Worth, Texas, USA) at the hydrogel-AFM probe interface.

### Scanning transmission electron microscopy (STEM) and scanning electron microscopy (SEM)

A FEI Quanta 250 field emission scanning electron microscope (FEG SEM) system equipped with a scanning transmission electron microscope (STEM) detector was used to image the lehfilcon A CL and SiHy base substrate samples. For sample preparation, the lenses were first rinsed in water and cut into pie-shaped wedges. In order to achieve a differential contrast between the hydrophilic and hydrophobic components of the samples, a 0.10% stabilized RuO_4_ solution was used as a staining agent, into which the samples were immersed for 30 min. The RuO_4_ staining of the lehfilcon A CL is not only important to achieve an enhanced differential contrast, but it also helps in preserving the structure of the branched polymer brushes in their original form, which is then visible in the STEM imaging. Subsequently, they were rinsed and dehydrated in a series of ethanol/water mixtures with ascending ethanol concentration. This was followed by embedding the samples in EMBed 812/Araldite epoxy resin, which was polymerized overnight at 70 °C. The sample blocks attained via resin polymerization were sectioned using an ultramicrotome, and the resulting thin sections imaged using the STEM detector at an accelerating voltage of 30 kV in low-vacuum mode. The same SEM system was used to conduct a detailed characterization of the PFQNM-LC-A-CAL AFM probe (Bruker Nano, Santa Barbara, California, USA). The SEM images of the AFM probe were captured under typical high-vacuum mode at an accelerating voltage of 30 kV. Images were obtained at different angles and magnifications, in order to record all the details regarding the shape and size of the AFM probe’s tip. Digital measurements were made for all tip dimensions of interest in the images.

### AFM imaging

A Dimension FastScan Bio Icon Atomic Force Microscope (Bruker Nano, Santa Barbara, California, USA) with the “PeakForce QNM in Fluid” mode was used for both imaging and nanoindentation of the lehfilcon A CL, SiHy base substrate and the PAAm hydrogel samples. For imaging experiments, a PEAKFORCE- HIRS-F-A (Bruker) probe with a nominal tip radius of 1 nm was used to capture high-resolution images of the samples at a scan rate of 0.50 Hz. All imaging was conducted under aqueous solutions.

### AFM nanoindentation probes

**Figure 1:**
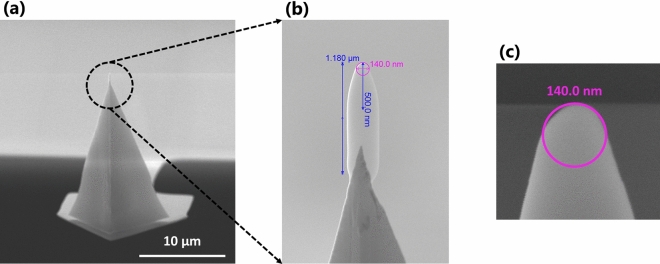
SEM images of the specially designed AFM probe used in the nanoindentation method.

The AFM nanoindentation experiments were conducted using the PFQNM-LC-A-CAL (Bruker) probe. This AFM probe has a silicon tip on a nitride cantilever, which is 345 nm thick, 54 µm long, 4.5 µm wide and, has a resonant frequency of 45 kHz, respectively. It is especially designed to characterize and perform quantitative, nano-mechanical measurements of soft biological samples. The probes are individually calibrated at the factory and come with a pre-calibrated spring constant. The spring constants of the probes used in this study were in the range of 0.05–0.1 N/m. In order to precisely determine the tip shape and dimensions, the probes were subjected to detailed characterization using the SEM. Figure 1a shows a high-resolution scanning electron micrograph of the PFQNM-LC-A-CAL probe at low magnification to provide an overall view of the probe design. A zoomed-in view of the very top part of the probe tip is shown in Fig. 1b, which provides information related to the tip shape and size. At its very end, the tip is a hemisphere with a measured diameter of about 140 nm (Fig. 1c). Below this, the tip gradually tapers into a conical shape extending to a measured length of about 500 nm. Beyond the conical region, the tip shape takes on a cylindrical form that ends at the total tip length of 1.18 µm. This represents the primary functional portion of the probe’s tip. In addition, a large spherical polystyrene (PS) probe (Novascan Technologies, Inc., Boone, Iowa, USA) with a tip diameter of 45 µm and a spring constant of 2 N/m was also used in testing as a control of a colloidal probe to compare with the 140 nm diameter PFQNM-LC-A-CAL probe.

### AFM nanoindentation experiment

It has been reported that liquids can be confined between the AFM probe and the polymer brush structure during nanoindentation, which will apply an upward force on the AFM probe before it actually touches the surface^69^. This viscous squeeze-out effect from the fluid confinement can shift the apparent contact point and thus impact the surface-modulus measurement. In order to examine the effect of probe geometry and indentation rate on the fluid confinement, indentation force-curves were generated for the lehfilcon A CL sample at constant displacement rates of 1 µm/s and 2 µm/s using the 140 nm diameter probe and at 1 µm/s rate for the 45 µm diameter probe, to a fixed force setpoint of 6 nN. The experiments carried out with the 140 nm diameter probe were conducted at an indentation rate of 1 µm/s and a force setpoint of 300 pN which were chosen to generate a contact pressure within the physiological range (1–8 kPa) of the upper eyelid pressure on the human ocular surface^72^. The soft, off-the-shelf 1 kPa PAAm hydrogel sample was tested to an indentation force of 50 pN at rate of 1 µm/s using the 140 nm diameter probe.

### Data fit model and processing

**Figure 2:**
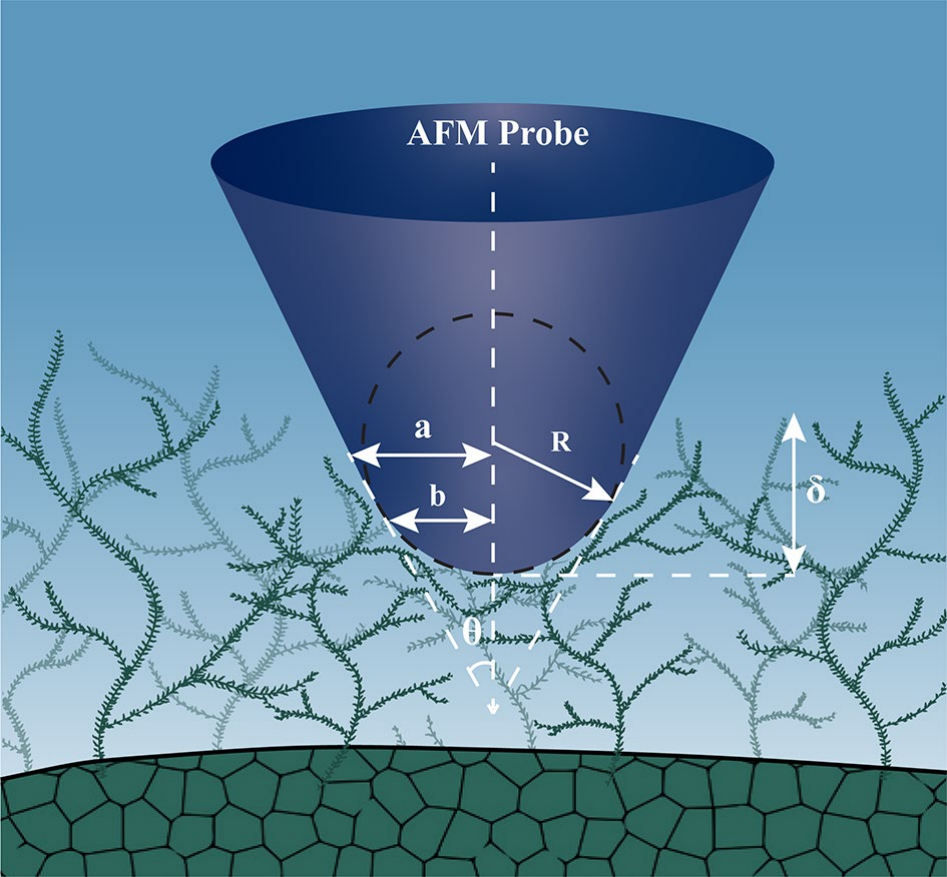
Schematic of the contact geometry of a rigid cone with a spherical tip indenting the lehfilcon A contact lens material with a branched polymer-brush surface layer.

As the length of the cone-sphere portion of the PFQNM-LC-A-CAL probe tip is ~ 500 nm, for any indentation with a depth < 500 nm, it is safe to assume that the geometry of the probe during indentation will stay true to its cone-sphere shape. In addition, it was assumed that the surface of the tested material will display reversible elastic response, which will also be shown to be true in the later section. Therefore, based on the shape and size of the tip, we selected the cone-sphere data fit model developed by Briscoe, Sebastian, and Adams, available in the vendor's software to process the force-separation data generated from our AFM nanoindentation experiments (NanoScope Analysis Software, Bruker)^73^. This model describes the force–displacement *F(δ)* relationship for a cone with spherical tip defect. Figure 2 shows the contact geometry used for the interaction of a rigid cone with a spherical tip indenting the contact lens material, where *R* is the radius of the spherical tip, *a* is the contact radius, *b* is the contact radius at the end of the spherical section, *δ* is the indentation depth, and *θ* is the half-included angle of the cone. The SEM images of this probe clearly show that the 140 nm diameter spherical tip tangentially transitions into the cone, thus *b* is defined here only in terms of *R*, that is, *b* = *R cos θ*. The vendor-supplied software provides a relation based on cone-sphere model to calculate the Young’s modulus (*E*) values from the force-separation data with an assumption of *a* > *b*. The relation is:

1 $$F =2\frac{E}{(1-{\nu }^{2})}\left[a\delta - \frac{{a}^{2}}{2tan\theta } \left[\frac{\pi }{2}-asin\left(\frac{b}{a}\right)\right]-\frac{{a}^{3}}{3R}+{\left({a}^{2}-{b}^{2}\right)}^{1/2}\left(\frac{b}{2tan\theta }+\frac{{a}^{2}-{b}^{2}}{3R}\right)\right]$$

where *F* is the indentation force, *E* is Young's modulus, and *ν* is Poisson's ratio. The contact radius, *a*, can be evaluated using:

2 $$\delta +\frac{a}{R}\left\{{\left({a}^{2}-{b}^{2}\right)}^{1/2}-a\right\}-\frac{a}{tan\theta }\left[\frac{\pi }{2}-asin\left(\frac{b}{a}\right)\right]=0$$

In case of *a* ≤ *b*, the relationship would have just reduced to the equation for usual spherical indenter;

3 $$P=\frac{4}{3}\frac{E}{\left(1-{\nu }^{2}\right)}\sqrt{R}{\delta }^{3/2}$$

We believe that the interaction of the indenting probe with branched PMPC polymer brush structure will be such that the contact radius *a* will be greater than that of the spherical contact radius *b*. Therefore, we have used the relations derived for case *a* > *b* for all the quantitative elastic modulus measurements performed in this study.

Other options used in the testing method and analysis were:

- Active Curve: Retract
- Fit Method: Contact Point Based
- Contact Point Algorithm: Best Estimate
- Contact Point: Based on background noise threshold
- Fit Model: Cone Sphere

## Results and discussion

### Surface morphology of the biomimetic material

**Figure 3:**
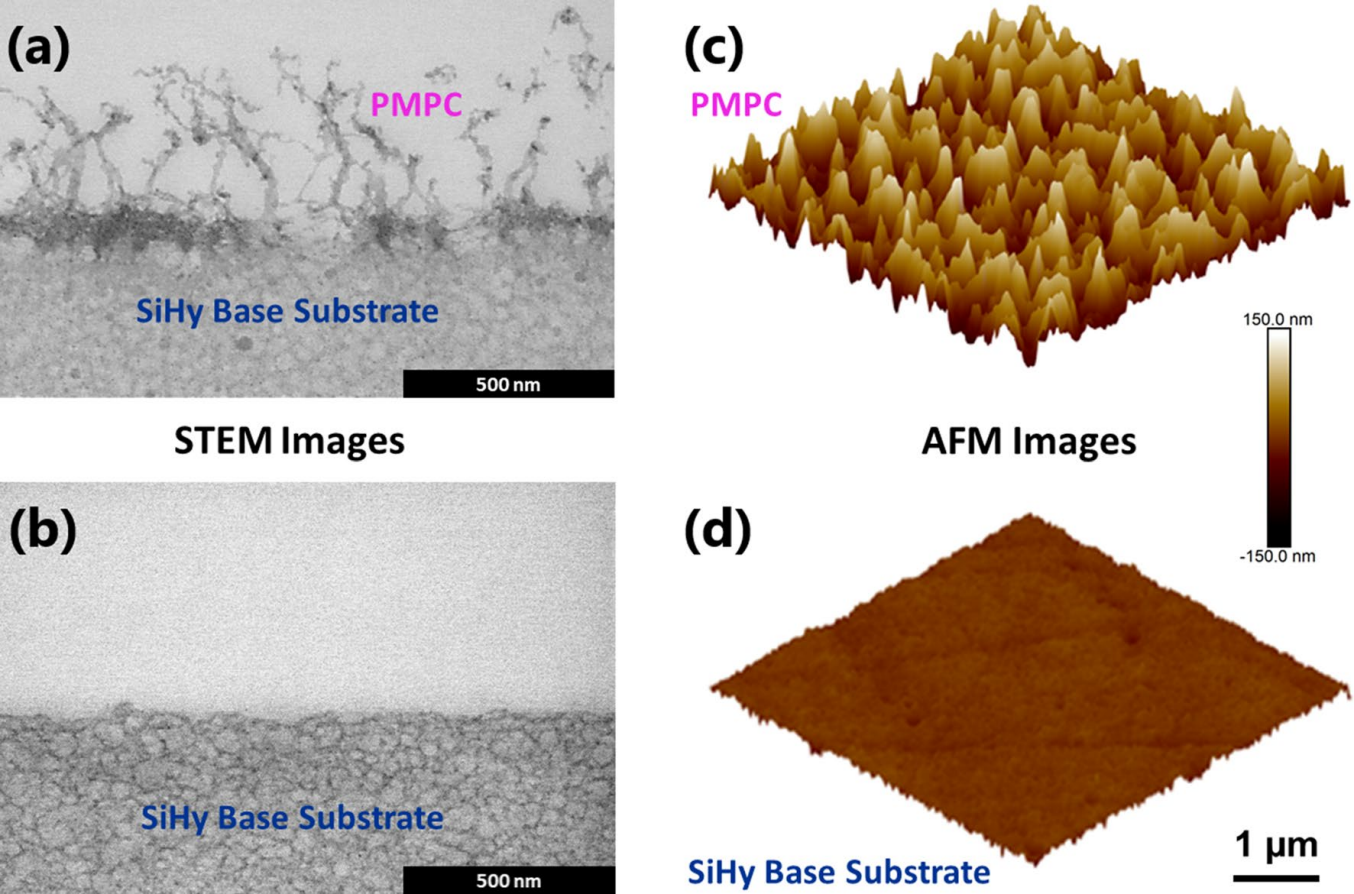
STEM images of cross-sections of (**a**) lehfilcon A CL and (**b**) SiHy Base Substrate. Scale bars, 500 nm. AFM images (3 µm × 3 µm) of the surfaces of (**c**) lehfilcon A CL and (**d**) SiHy Base Substrate.

Scanning transmission electron microscopy (STEM) of sample cross-sections and atomic force microscopy (AFM) of surfaces were employed to conduct a comprehensive imaging of the ultra-soft biomimetic materials examined in this study. This detailed surface characterization was carried out as an extension of our previously published work, where we established that the dynamic branched, polymer-brush structure at the surface of the PMPC-modified lehfilcon A CL exhibits mechanical properties similar to natural corneal tissue^14^. For this reason, we refer to the contact-lens surface as a biomimetic material^14^. Figure 3a,b show cross-sections of the branched PMPC polymer-brush structure on the lehfilcon A CL and the surface of the untreated SiHy base substrate, respectively. The surfaces of both samples were further analyzed using high-resolution AFM imaging techniques, which further confirmed the findings of the STEM analysis (Fig. 3c,d). Collectively, these images provided an approximate length of the branched PMPC polymer brush structure of 300–400 nm, which is critical for the interpretation of the AFM nanoindentation measurements. Another key observation derived from the imaging is that the overall structure at the surface of the biomimetic CL material is morphologically different from that of the SiHy base substrate material. This difference in their surface morphologies could become evident in their mechanical interaction with the indenting AFM probe and subsequently in the measured modulus values.

### Atomic force microscopy (AFM) nanoindentation testing method

Bioinspired polymers and polymer-brush structures, which are inherently very soft, have been explored and used extensively in several biomedical applications^74–77^. Therefore, it is very important to use AFM nanoindentation methods that can accurately and reliably measure their mechanical properties. However, at the same time, the unique properties possessed by these ultra-soft materials, e.g., extremely low modulus, high fluid content, and high elasticity, often make it difficult to make the right choice for the indenting probe material, shape, and size. This is critical in assuring that the indenter does not pierce through the soft sample surface, leading to errors in the determination of the point of surface contact and contact area.

**Figure 4:**
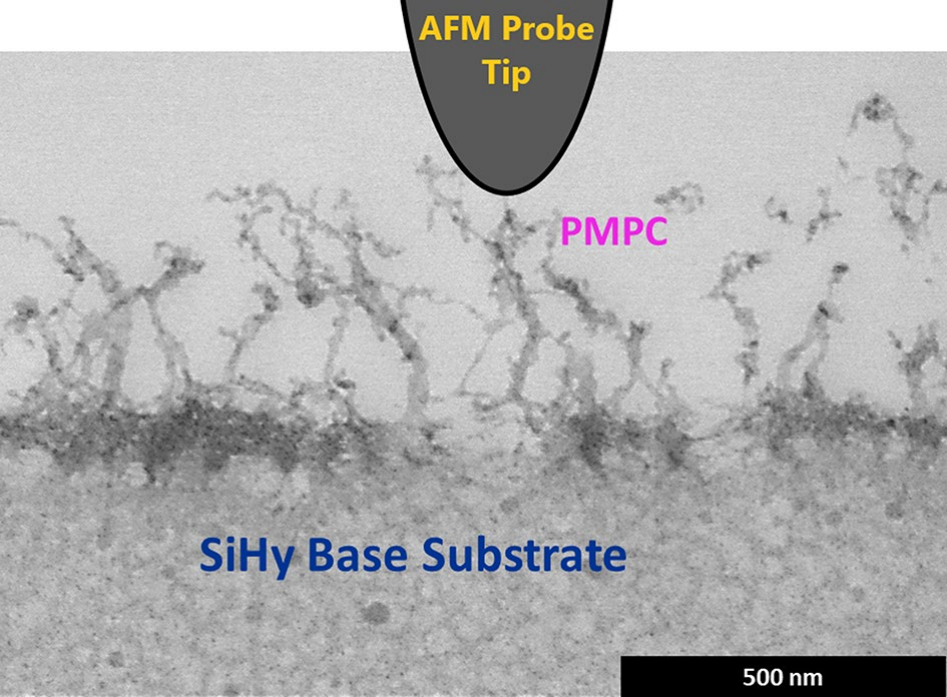
Schematic showing the STEM image of a lehfilcon A CL and the indenting AFM probe tip (drawn to scale).

To this end, it was important to have a comprehensive understanding of the morphology of the ultra-soft biomimetic material (lehfilcon A CL). The information regarding the branched polymer brush size and structure provided by the imaging techniques laid the framework for the surface-mechanical characterization using the AFM nanoindentation method. Instead of using a micrometer-size spherical colloidal probe, we selected a silicon nitride PFQNM-LC-A-CAL (Bruker) probe with a 140 nm tip diameter, especially designed to perform quantitative mapping of the mechanical properties of biological samples^78–84^. The rationale behind using a relatively sharp probe compared to the traditional colloidal probe can be explained using the structural characteristics of the material. By comparing the size of the probe tip (~ 140 nm) to the branched polymer brush features at the surface of the lehfilcon A CL shown in Fig. 3a, the tip end is sufficiently large to make a direct contact with these brush structures, thus reducing the possibility of the tip piercing through them. To illustrate this, a diagram of the STEM image of a lehfilcon A CL and the indenting AFM probe tip (drawn to scale) is provided in Fig. 4.

**Figure 5:**
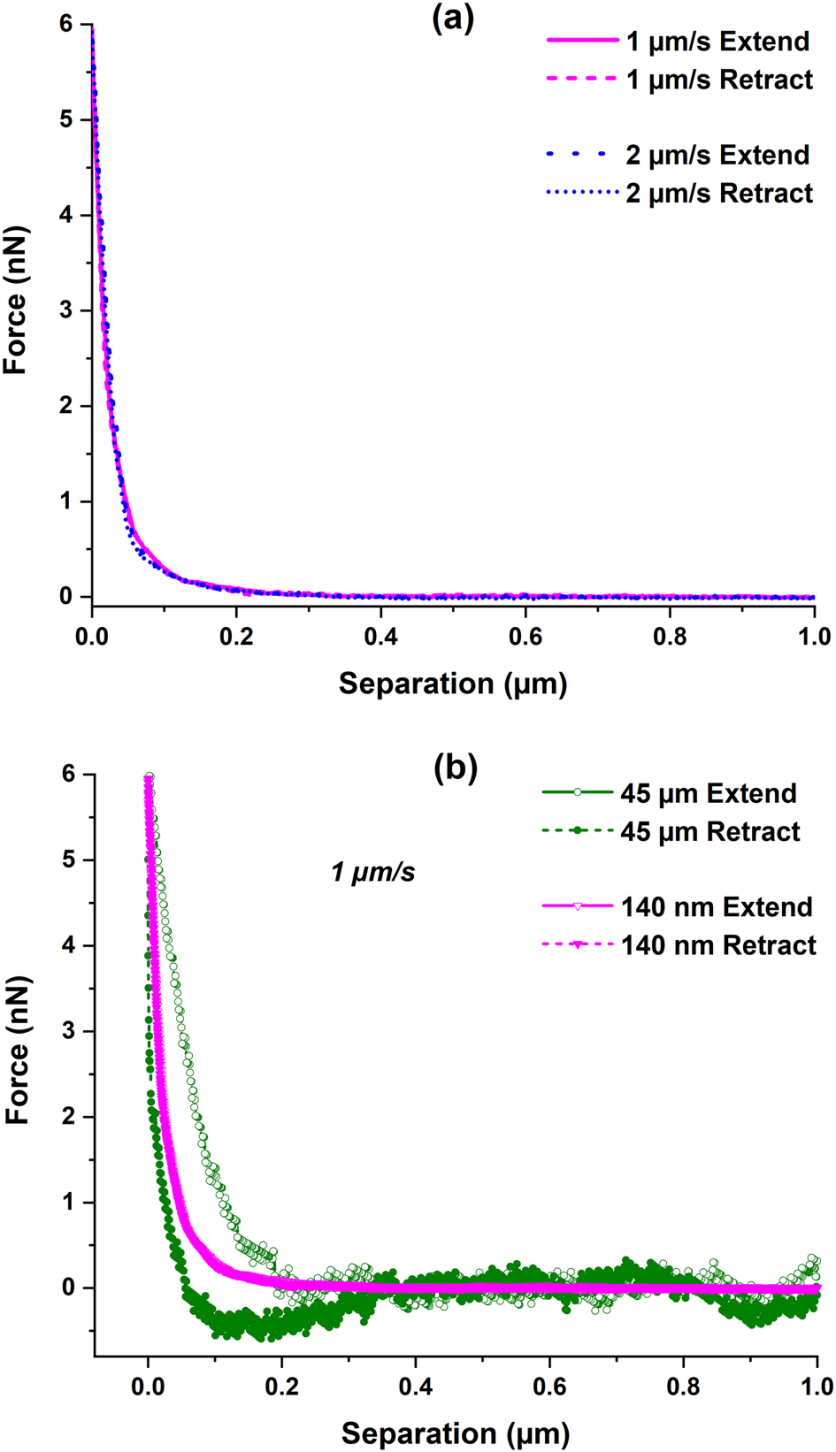
AFM indentation force-curves for lehfilcon A CL; (**a**) using the 140 nm diameter probe at two loading rates showing the absence of poroelastic effects during surface indentation; (**b**) using 45 µm and 140 nm diameter probes at 1 µm/s rate showing the presence of both viscous squeeze-out and poroelastic effects for the larger probe compared to the smaller one.

In addition, the 140 nm tip size is small enough to avoid the risk of any viscous squeeze-out effect that has been previously reported for the polymer brushes indented using a CP-AFM nanoindentation method^69,71^. We hypothesized that due to the special cone-sphere shape and relatively small dimensions of this AFM tip (Fig. 1), the nature of the force-curves resulting from the nanoindentation of the lehfilcon A CL would be independent of the indentation speed or the loading/unloading rate and therefore unaffected by poroelastic effects. In order to test this hypothesis, the PFQNM-LC-A-CAL probe was used to indent the lehfilcon A CL sample to a fixed maximum force but at two different speeds, and the resulting extend and retract force-curves were used to generate the force (nN) vs. separation (µm) plot shown in Fig. 5a. Clearly, there is a complete overlap between the force-curves during loading and unloading, and there is no visible evidence of increasing force offset at zero indentation depth with an increasing rate of indentation in the plot^69^, suggesting that it is the individual brush elements that have been characterized, in absence of poroelastic effects. In contrast, fluid confinement effects (viscous squeeze-out and poroelastic effects) are clearly evident for the 45 µm diameter AFM probe at same indentation rate and are highlighted by the presence of hysteresis between the extend and retract curve as shown in Fig. 5b. These results support the hypothesis and suggest that the 140 nm diameter probe is a good choice to characterize such soft surface features.

**Figure 6:**
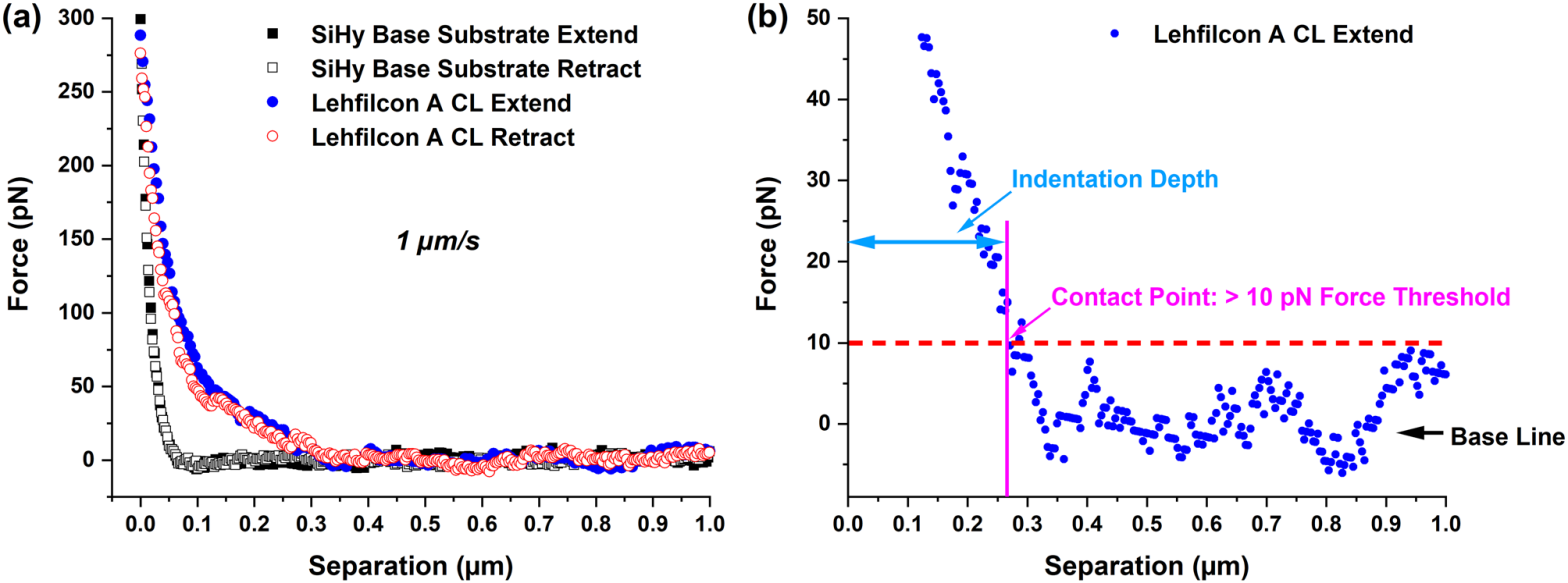
(**a**) AFM nanoindentation force-curves for lehfilcon A CL and SiHy base substrate; (**b**) force curve showing contact point estimation using the background noise threshold approach.

To characterize an ultra-soft surface, an AFM nanoindentation method must have a probe that is best suited to the nature of the material to be examined. In addition to the shape and size of the probe, the sensitivity of the AFM detector system, deflection sensitivity of the probe in the testing medium, and the cantilever’s spring constant all play major roles in determining the accuracy and reliability of the nanoindentation measurements. For our AFM system, the detection limit of the position-sensitive detector (PSD) was about 0.5 mV and based on the PFQNM-LC-A-CAL probe’s pre-calibrated spring constant and the calculated deflection sensitivity in fluid, this corresponds to a theoretical load sensitivity of the method of less than 0.1 pN. Thus, in the absence of any peripheral noise component, the method has the capability to measure a minimum indentation force of ≤ 0.1 pN. However, it is almost impossible for the AFM system to reduce the peripheral noise to this level due to factors such as mechanical vibration and hydrodynamic forces. These factors limit the overall sensitivity of the AFM nanoindentation method and also contribute to a background noise signal of about ≤ 10 pN. In order to perform the surface characterization, the lehfilcon A CL and SiHy base substrate samples were indented using a SEM-characterized, 140 nm diameter probe under fully hydrated conditions, and the resulting force curves were then superimposed in the force (pN) vs. separation (µm) plot shown in Fig. 6a. When compared to the SiHy base substrate, the force curves for the lehfilcon A CL clearly show a transition phase beginning at the point of contact with the branched polymer brushes and ending with a sudden change in the slope, marking the tip contact with the underlying base material. This transitory section of the force-curve highlights the truly elastic behavior of the branched polymer brushes at the surface, as evidenced by the retract curve perfectly following the extend curve, and also the contrast in mechanical properties between the brush structures and the bulk SiHy material. By comparing the average length of the branched polymer brushes in the STEM image (Fig. 3a) of the lehfilcon A CL with the separation values for its force curves on the x-axis in Fig. 6a, it is clear that the method was able to register the contact between the tip and the branched polymer brush structures extending to the very top of the surface. In addition, the close overlap of the force curves points to the absence of any fluid confinement effect taking place. At the same time, adhesive forces between the tip and the sample surface were completely absent. The top-most sections of the force-curves for both samples overlap, reflecting the similarities in the mechanical properties of the base substrate material.

To explore the finer details of the force curves, the extend curve for the lehfilcon A CL sample is re-plotted in Fig. 6b with the maximum force value on the y-axis at 50 pN. This plot provides important information regarding the baseline background noise. The noise is in range of ± 10 pN, and this value was used to perform precise contact point determination and indentation depth calculations. It is well reported in the literature that identification of contact point is crucial for accurate estimation of material properties such as elastic modulus^85^. A method involving automated processing of force-curve data has shown improved consistency for data fitting and quantitative measurements for soft materials^86^. In this work, our choice of contact point is relatively straightforward and objective but with its own limitations. Our conservative approach of contact point determination could lead to slight over-estimation of modulus values for the shallower indentation depths (< 100 nm). The use of algorithm-based contact point determination and automated data processing could be an extension of this work in the future, for further improvement of our method. Therefore, with respect to the inherent baseline background noise being of the order of ± 10 pN, we have defined the contact point as the first data point on the x-axis in Fig. 6b which has a value ≥ 10 pN. Then, based on the 10 pN noise threshold, the vertical line at ~ 0.27 µm marks the surface contact point, after which the extend curve continues until the substrate is encountered at an indentation depth of ~ 270 nm. Interestingly, based on the size of the branched polymer brush (300–400 nm) features measured using the imaging techniques, the indentation depth of ~ 270 nm observed for the lehfilcon A CL sample using the background noise threshold approach stands remarkably close to the dimensions measured by STEM. These findings further confirm the compatibility and suitability of the AFM probe’s tip shape and size for the indentation of this the very soft and highly elastic branched polymer brush structure. The data also provide strong evidence in support of our approach for precise contact-point determination using the background noise as the threshold value. Consequently, any quantitative results deduced from mathematical modeling and fitting of the force-curves should be relatively accurate.

**Figure 7:**
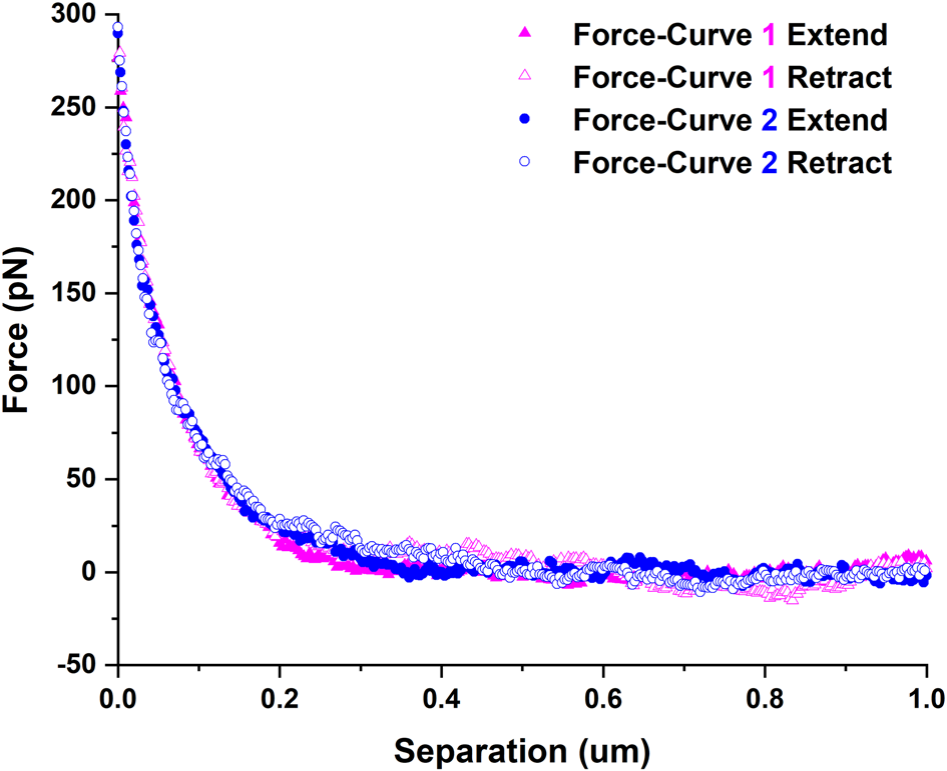
Two indentation force-curves at the same location of the lehfilcon A CL surface indicating the perfectly elastic nature of the lens surface.

Quantitative measurements from an AFM nanoindentation method are completely dependent on the mathematical model used to perform the data fitting and subsequent analysis. Therefore, it is very important that all the factors related to the choice of the indenting probe, the material properties and the mechanics of their interaction are taken into consideration before choosing a particular model. In this case, in-depth characterization of the tip geometry was conducted using the SEM micrographs (Fig. 1), and based on the results, the 140 nm diameter AFM nanoindentation probe with the geometry of a rigid cone and a spherical tip is a good choice for characterizing the lehfilcon A CL sample^79^. Another important factor that needs to be thoroughly assessed is the elastic properties of the polymeric material being tested. Even though the initial nanoindentation data (Figs. 5a and 6a) clearly depicted the overlapping characteristics of the extend and the retract curves, i.e., full elastic recovery of the material, it is crucial to confirm the purely elastic nature of the contact. To that end, two successive indentations were carried out at the same location on the surface of lehfilcon A CL sample at a 1 µm/s indentation rate in fully hydrated conditions. The resulting force-curve data is presented in Fig. 7, and as expected, the extend and the retract curves from both the indentations are virtually identical, thus highlighting the high elasticity of the branched polymer brush structures.

Based on the information from the SEM and STEM images of the probe tip and the lehfilcon A CL surface, respectively, the cone-sphere model is a reasonable mathematical representation of the interaction between our AFM probe tip and the tested soft polymeric materials. In addition, for this cone-sphere model, the fundamental assumption of the elastic nature of the indented material is valid in the case of this novel biomimetic material and was used for quantitative elastic modulus estimations.

### Surface-modulus measurements

**Figure 8:**
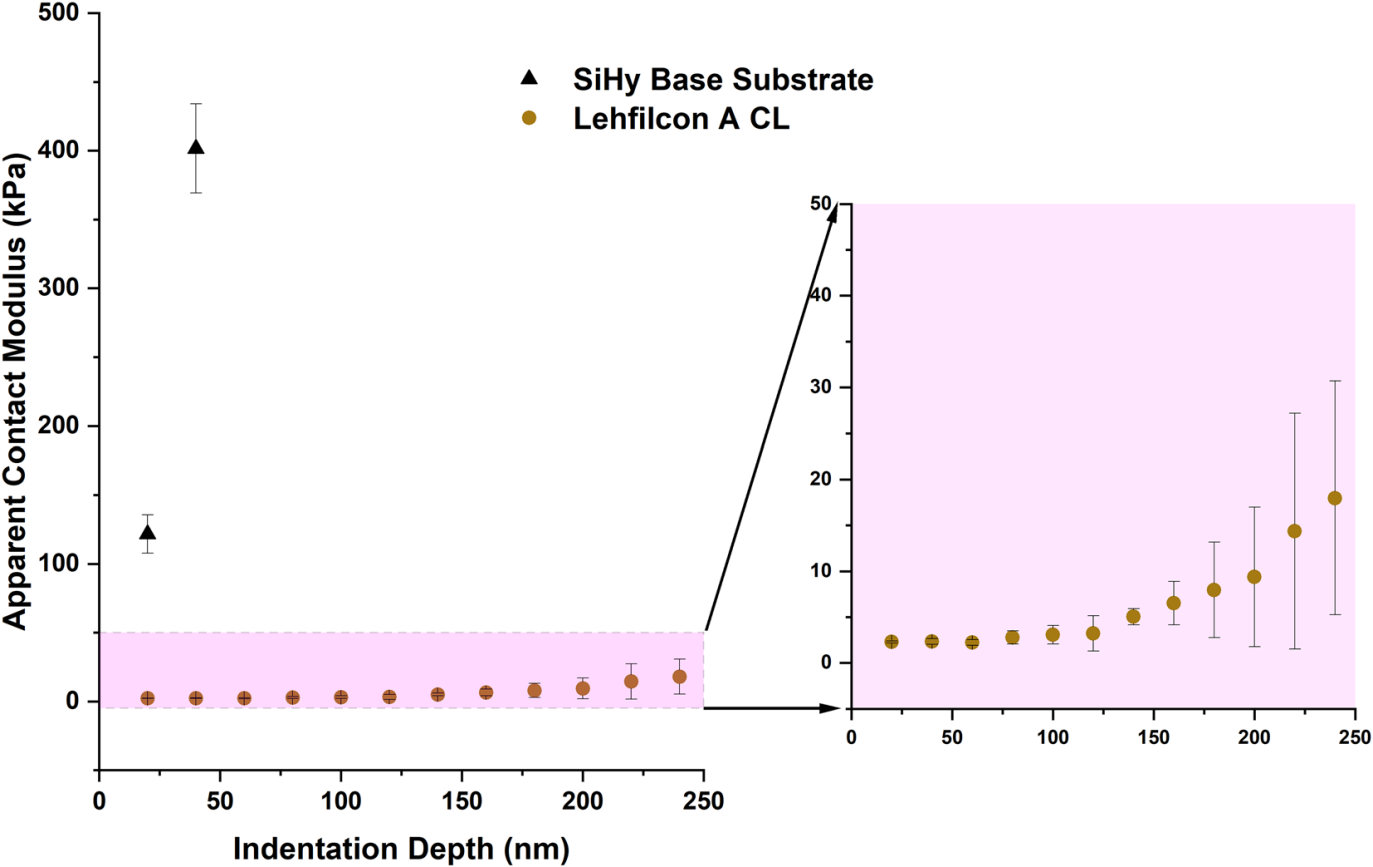
Apparent contact modulus (kPa) as a function of indentation depth (nm) for the lehfilcon A CL and the SiHy base substrate using the AFM nanoindentation method employing the cone-sphere geometry model for modulus measurement.

After comprehensive assessment of the AFM nanoindentation method and its components, including indenting-probe characteristics (shape, size, and spring constant), sensitivity (background noise and contact-point estimation) and the data-fit model (quantitative modulus measurement), the method was used to characterize a commercially available ultra-soft sample in order to validate the quantitative results. A commercial polyacrylamide (PAAm) hydrogel with an elastic modulus of 1 kPa was tested with the 140 nm diameter probe under hydrated conditions. Details regarding the tests and modulus calculations are provided in the Supplementary Information. The results showed that the average measured modulus is 0.92 kPa, and the %RSD and percent (%) deviation from the known modulus are both less than 10 percent. These results verify the accuracy and reproducibility of the AFM nanoindentation method used in this work to measure the modulus of the ultra-soft materials. The surfaces of the lehfilcon A CL and the SiHy base substrate samples were further characterized using the same AFM nanoindentation method to examine the ultra-soft surface’s apparent contact modulus as a function of the indentation depth. Indentation force-separation curves were generated for three samples of each kind (n = 3; one indentation per sample) at a 300 pN force setpoint, 1 µm/s rate, and in fully hydrated conditions. The indentation force-separation curves were fitted using the cone-sphere model. To derive an indentation-depth-dependent modulus, sections of the force-curve of 40 nm width were fitted at each incremental step of 20 nm, starting from the point of contact, and modulus values measured at each step on the force curve were recorded. Spencer et al. have used a similar approach to characterize modulus gradients of poly (dodecyl methacrylate) (P12MA) polymer brushes using colloidal-probe AFM nanoindentation, where they fitted the data using the Hertzian contact model^69^. This approach provided the apparent contact modulus (kPa) vs indentation depth (nm) curves shown in Fig. 8, which illustrates the apparent contact modulus/depth gradient. The calculated elastic modulus for the lehfilcon A CL sample within the top 100 nm of the sample is in the range of 2–3 kPa, and beyond this point it starts to increase with increasing depth. On the other hand, in testing the SiHy base substrate without a brush structured film on its surface, the maximum indentation depth achieved at the 300 pN force was less than 50 nm, and the modulus value derived from the data was ~ 400 kPa, comparable to the Young’s modulus value of the bulk material.

The very top surface of the novel, biomimetic, branched polymer-brush structure exhibits an extremely low modulus (2–3 kPa). This would correspond to the free-dangling ends of the branched polymer brushes, as visible in the STEM images. While there is some evidence of a gradient in modulus at the outer edge of the CL, a greater effect is that of the high-modulus substrate below. However, the top 100 nm of the surface is within the 20% of the total length of the branched polymer brushes, so it is reasonable to believe that the modulus values measured within this range of indentation depth are relatively accurate and are not strongly influenced by substrate effects.

## Conclusions

Since the lehfilcon A contact lens has a unique biomimetic design consisting of branched PMPC polymer brush structures grafted onto the surface of a SiHy base substrate, it is very challenging to reliably characterize the mechanical properties of its surface structure using traditional measurement techniques and methods. Here, we have presented an improved AFM nanoindentation method that can accurately characterize ultra-soft materials such as lehfilcon A that have high water content and very high elasticity. This approach relies on the use of an AFM probe with a tip size and geometry carefully chosen to match the structural dimensions of the ultra-soft surface features to be indented. This size pairing between the probe and the structure provided enhanced sensitivity that allowed us to measure the low modulus and intrinsic elastic properties of the branched polymer brush elements without the influence of poroelastic effects. The results showed that the unique, branched PMPC polymer-brush features at the lens surface have an extremely low elastic modulus (as low as 2 kPa) and very high elasticity (nearly 100%) when tested in an aqueous environment. The AFM nanoindentation results also enabled us to characterize the apparent contact modulus/depth gradient (30 kPa/200 nm) for the biomimetic lens surface. This gradient could be resulting from the modulus difference between the branched polymer brushes and the SiHy substrate or due to the branching structure/density of the polymer brushes or from a combination of both. Nevertheless, further in-depth research is needed to understand the structure–property correlation completely, and in particular the role of brush branching on mechanical properties. A similar measurement approach may prove useful in characterizing the surface mechanical properties of other ultra-soft materials and medical devices.

## Supplementary Information

Supplementary Information.

## Data Availability

The datasets generated during and/or analyzed during the current study are available from the corresponding author on reasonable requests.
