# Supplementary material for: Surface characterization of an ultra-soft contact lens material using an atomic force microscopy nanoindentation method
Source: Sci Rep. 2022 Nov 21;12:20013. doi: 10.1038/s41598-022-24701-9 (PMC9678857; doi:10.1038/s41598-022-24701-9)
Supplement: Supplementary file 1 — Supplementary Information. [file 41598_2022_24701_MOESM1_ESM.pdf]

Surface Characterization of an Ultra-Soft Contact Lens Material Using an Atomic Force Microscopy Nanoindentation Method

Vinay Sharma[1], Xinfeng Shi[1], George Yao[2], George M. Pharr[3], James Yuliang Wu[1*]

[1]Alcon Research, LLC, Fort Worth, TX 76134 USA

[2]Alcon Research, LLC, Duluth, GA 30097 USA

[3] Department of Materials Science and Engineering, Texas A&M University, College Station, TX 77843 USA

*Corresponding Author: J. Y. Wu

E-mail: james.wu@Alcon.com

Supplementary Information

Nanoindentations with the 140 nm diameter probe at ~50 pN maximum force were generated at three different locations on the hydrogel's surface, and the cone-sphere data fit model was used to calculate the modulus values. Supplementary Table S1 displays the calculated elastic moduli for the 1 kPa PAAm hydrogel sample, their average, standard deviation, percent relative standard deviation (% RSD) and the percent (%) deviation from the given modulus value provided by the manufacturer.

Supplementary Table S1. Elastic modulus data for the 1 kPa PAAm hydrogel standard using the cone sphere model.

Given Modulus (kPa)	AFM Nanoindentation at ~50 pN: Calculated Modulus (kPa)			Average Modulus (kPa)	SD	% RSD	% Deviation from Given Modulus
	1	2	3				
1	1.02	0.88	0.85	0.92	0.09	9.94	8.33
